# Data Collection Framework for Context-Aware Virtual Reality Application Development in Unity: Case of Avatar Embodiment

**DOI:** 10.3390/s22124623

**Published:** 2022-06-19

**Authors:** Jiyoung Moon, Minho Jeong, Sangmin Oh, Teemu H. Laine, Jungryul Seo

**Affiliations:** 1 Department of Digital Media, Ajou University, Suwon 16499, Korea; jiyoungmoon55@gmail.com (J.M.); minho6428@naver.com (M.J.); ohsm1226@naver.com (S.O.); 2 Smart Mobility Lab, B2B Advanced Technology Center, LG Electronics, Seoul 07796, Korea; jrseojr@naver.com

**Keywords:** Virtual Reality, Unity, context data collection, framework, embodiment, context-awareness, metaverse, formative evaluation

## Abstract

Virtual Reality (VR) has been adopted as a leading technology for the metaverse, yet most previous VR systems provide one-size-fits-all experiences to users. Context-awareness in VR enables personalized experiences in the metaverse, such as improved embodiment and deeper integration of the real world and virtual worlds. Personalization requires context data from diverse sources. We proposed a reusable and extensible context data collection framework, ManySense VR, which unifies data collection from diverse sources for VR applications. ManySense VR was implemented in Unity based on extensible context data managers collecting data from data sources such as an eye tracker, electroencephalogram, pulse, respiration, galvanic skin response, facial tracker, and Open Weather Map. We used ManySense VR to build a context-aware embodiment VR scene where the user’s avatar is synchronized with their bodily actions. The performance evaluation of ManySense VR showed good performance in processor usage, frame rate, and memory footprint. Additionally, we conducted a qualitative formative evaluation by interviewing five developers (two males and three females; mean age: 22) after they used and extended ManySense VR. The participants expressed advantages (e.g., ease-of-use, learnability, familiarity, quickness, and extensibility), disadvantages (e.g., inconvenient/error-prone data query method and lack of diversity in callback methods), future application ideas, and improvement suggestions that indicate potential and can guide future development. In conclusion, ManySense VR is an efficient tool for researchers and developers to easily integrate context data into their Unity-based VR applications for the metaverse.

## 1. Introduction

As global interest toward Virtual Reality (VR) is increasing, there have been attempts to utilize VR in various fields such as second chance tourism [1], mental health therapy [2], and education [3]. The recent proliferation of VR applications and services can be partly explained by affordable head-mounted displays (HMDs), such as Meta Quest 2 and HTC VIVE, that are capable of delivering highly immersive interaction experiences to users. At the same time, popular game development platforms, such as Unreal Engine and Unity, have contributed to the growing popularity of VR technology through provision of powerful development tools for creating VR applications.

VR experiences are traditionally built upon VR equipment comprising a HMD and hand-worn motion-tracking controllers that enable the user to be visually and aurally immersed in a virtual environment. However, a shortcoming of this approach is that it typically provides one-size-fits-all experiences to users with little consideration for their diverse contexts. Thus, context-aware VR—which reaches beyond the basic sensing capabilities of VR hardware—has emerged as an interesting research thread that attempts to bring the affordances of context-aware computing (e.g., high personalization) into virtual worlds. However, so far the number of studies exploring context-aware VR has been limited [4].

Several approaches can be considered for creating a system that understands the user’s context. A common approach is classifying the user’s context using sensor data captured from the user. For instance, Seo et al. developed emotion classification models using the user’s electroencephalogram (EEG) data and machine learning classifiers to be applied to VR contents for Alzheimer’s disease patients [5]. Seo et al.’s study used only one sensor device; however, a key aspect of context-aware VR systems is to support data collection from diverse sensor sources because this way the system can be more aware of the user’s context [6]. For example, researchers have connected wearable physiological sensors related to heart and brain activity to a virtual environment to collect physiological data to be used in a VR experience [7,8]. In previous context-aware VR systems, the method of collecting data from sensors has typically been tightly integrated into a VR project so that it is not easy to adapt the same data collection method to other projects [2,7]. Consequently, a context data collection framework that can be easily harnessed in various types of virtual environments is needed to speed up the development of context-aware VR applications.

Embodiment is an important part of immersive VR experiences and it is one of the contributors to the feeling of presence experienced by users of VR [9]. Kilteni et al. [10] define the sense of embodiment in VR as an “ensemble of sensations that arise in conjunction with being inside, having, and controlling a body”. In the context of VR, the body is typically that of an avatar that is synchronized with the user’s body; the user controls the avatar with their bodily gestures, such as movement of limbs and head. To implement rich embodiment, accurate tracking of the user’s body is required; this can be performed with sensors that track different body parts (e.g., head, hands, fingers, eyes, face, and so forth). In other words, embodiment is an application of context-awareness that requires the VR system to acquire diverse context data from multiple sources and then process the data to replicate the user’s bodily actions in the body of an avatar.

This work is inspired by ManySense, a sensor data collection and processing middleware for facilitating context-aware application development on Android devices [11]. ManySense was able to collect sensor data from both physical and virtual sensors that were connected to the mobile device by various methods, such as Bluetooth and WiFi. Additionally, it supported integrated context inference modules to enable further analysis of collected data. Drawing from the concept of ManySense, the objective of this study is to design and implement an efficient context data collection system to facilitate development of context-aware VR applications. We aim to reach this objective by proposing a Unity-based framework—ManySense VR—that separates the responsibility of data collection from each sensor to a dedicated manager component, thus enabling easy adding or removing of sensors by the developer. Specifically, the study has the following contributions: (i) Implementation of ManySense VR as an extensible context data collection framework that is capable of gathering data from diverse sources and that can be reused in different Unity-based VR projects; (ii) demonstration of the framework’s operation through an implementation of rich embodiment in a VR scene that uses ManySense VR to acquire data from the user’s bodily actions; (iii) technical evaluation of ManySense VR to demonstrate its effects on resource use; and (iv) qualitative formative evaluation involving five developers who provided their insights on ManySense VR as a development tool after using and extending it.

## 2. Background

### 2.1. Context-Awareness in VR

In this paper, we adopt the definition of context by Abowd et al. [12], who defined it as “any information that can be used to characterize the situation of an entity, where an entity can be a person, place, or physical or computational object.” Moreover, the general definition of context-awareness is the transferring of the user’s real-life information to the computer in order to use the context data to provide context-sensitive information or services to the user. A typical example of this are location-based games that use the user’s current location information to provide meaningful location-sensitive content [13]. In another example, Raj et al. [14] proposed the concept of contextual frames as abstractions of the user’s recurring contexts that can be relevant to the total outcome of the user’s behavior in a context-aware health self-management system. In their system, users and caregivers provided regular reports on users’ contextual data related to their physical activity, eating, and sleeping. They found that the same contextual factors—such as physical activity, food, and nearby people—can have different influences in different contextual frames. Because each user’s contextual frames are different, it is meaningful for the designer to consider the diverse contexts of users and context-awareness of the system in the design process [14].

According to the aforementioned definition of context, most VR systems are essentially context-aware as they follow the player’s movement in order to move the camera (HMD) and virtual hands (controllers). However, it is important to improve context-awareness in VR systems beyond this to facilitate the blending of the physical world and virtual world in the metaverse [15]. Moreover, a higher degree of context-awareness creates opportunities for personalization of VR experiences. For example, high quality embodiment, which is elaborated in the next section, requires accurate tracking of the user’s body beyond head and hands. Additionally, in various papers, researchers tried to improve context-awareness in VR in many ways, and their methods depend on the goal of the system. In the context of VR applications, Yang et al. [16] investigated position synchronization in a multi-user environment comprising both VR and augmented reality (AR) systems. In their system, both VR users’ and AR users’ positions were calibrated and synchronized so that they could feel co-located in the same space. The results suggest that conversation between users might become interactive and intuitive, whilst the work efficiency of workers might be enhanced by letting them understand the integration of spatial, auditory, and visual cues [16]. Another study found that an alert-based spatial notification system for HMD users helps them understand real-world places better in a situation where the virtual environment is isolated from the real world [17]. Finally, Yigitbas et al. [4] proposed adaptive interaction techniques for VR based on context data acquired from the user, platform, and environment. The system used a rule-based engine to analyze the context data and consequently adapt the user interface in terms of layout, modality, navigation, and task features.

To become context-aware, a VR system needs to collect context data from diverse sources. However, data collection can be error-prone and cumbersome, as different sensor devices and other data sources use various data acquisition methods that differ in many ways, such as connection method (e.g., serial connection, Representational State Transfer (REST) connection), programming language, sampling rate, data format, and communication model (polling or pushing). Therefore, it is desirable to have a reusable context data collection system that can be utilized in different VR projects, and that provides an easy way for a developer to extend upon. Our analysis of previous studies on context-awareness in VR suggests that there is a need for a reusable framework for providing context data that VR applications can utilize to improve the delivery of context-aware services. Thus, we focus on facilitating context-awareness in VR by proposing a context data collection framework for collecting signals from diverse data sources.

### 2.2. Embodiment in VR

In this study, we explore embodiment as a case context-awareness. In this section, we aim to gain an understanding on how other researchers have implemented embodiment in VR by reviewing previous studies on embodiment experiments. Previous studies utilized various sensors that track the user’s movement and actions to provide real-time body synchronization for the purpose of embodiment in VR. For example, Gall et al. [18] selected the Unity platform to implement a VR experience with embodiment and emotional stimuli. They chose the HTC VIVE VR apparatus including a head-mounted display, controllers, and motion trackers for tracking the user’s movement and synchronizing it with the avatar’s movement. This kind of setup that is built on off-the-shelf hardware and software is rather common in VR embodiment implementations. In another study, Krekhov [19] used an inverse kinematics (IK) method for generating virtual avatar poses depending on motion trackers’ positions and rotations. For high quality synchronization and specific detailed embodiment for expressing diverse human joint movements, Kim [20] used a full-body motion-capture system (Motive 2.0.2; NaturalPoint, Oregon) with Flex13 cameras (NaturalPoint, Oregon) and thirty-seven markers on a motion capture suit [20]. The positions of markers were used for mapping the user’s avatar in a tracked area by a camera. This kind of complex motion-capture setup is typically more expensive and more difficult to use than the motion capture provided by a VR hardware. The degree of embodiment varies between implementations. According to Gao et al. [21], users feel more comfortable when the avatar body is fully synchronized rather than just showing their hands [21]. They further argued that the user’s feeling of embodiment and presence can be increased when their virtual body is expressed more accurately. However, interestingly, Gao et al. [21] found that the absence of the lower body did not significantly affect the user’s embodiment experience; they noticed that the lower body was often ignored even when the avatar had a lower body.

A recent study measured users’ emotional responses according to the presence or absence of virtual embodiment [18]. The results suggested that users showed more emotional responses when embodiment is present. There are various reasons as to why the user can exhibit enhanced emotional responses to virtual embodiment. For example, Wiederhold found that rather than playing two roles of imagining and empathizing while listening to a story, it is easier to empathize with a specific virtual environment background [22]. Some aspects of technological performance can also affect the virtual experience quality and embodiment. According to Roth and Latoschik [23], embodiment experience can be negatively influenced by the degree of latency and jitter in network transmission. This is particularly important in VR-based metaverse systems where large numbers of simultaneous users coexist in the same virtual world as embodied avatars. Gonzalez-Franco et al. [24] found another method to improve the embodiment experience in VR. They said allowing the user to look at a virtual mirror and thereby feel ownership for the virtual avatar improves the feeling of embodiment in VR. Through this body ownership illusion, the user can feel the effect that the virtual body seems to be their body, which can increase the immersion in the virtual world. Krekhov et al. [19] demonstrated that the body ownership through embodiment in a virtual world can also be applied to animal bodies.

Based on our analysis of previous research on embodiment in VR environments, we found that implementing embodiment in VR often relies on various sensors, and there are many important factors that affect embodiment, such as network conditions, the extent of embodiment, and the existence of a virtual mirror. This information helped us build a research background to design a rich embodiment scene in VR to demonstrate the use of context data acquired through the ManySense VR context data collection framework.

## 3. ManySense VR

To facilitate the development of context-aware VR applications on the Unity platform, we designed the ManySense VR context data collection framework so that it can be easily extended with new data sources. ManySense VR was created as a collection of Unity scripts, thus allowing VR developers of this popular game engine to harness the power of context data in their applications. Its implementation relies on a set of application programming interfaces (APIs) provided by sensor device manufacturers and other data providers. In the following sections, we outline the details of ManySense VR, starting with the context data sources used and followed by the framework’s architecture.

### 3.1. Context Data Sources

Our motivation to develop ManySense VR was to provide rich embodiment experience in VR by collecting diverse context data related to the user’s body—both inside and outside. Therefore, we chose a set of sensor devices that detected not only movement of the user’s head, arms, and chest, but also eyes, lips, and jaw, as well as physiological data that could further enhance the embodiment experience through real-time data visualization. Moreover, during the formative evaluation, we acquired data from the Open Weather Map API as a demonstration of the extensibility of ManySense VR beyond sensor devices. Table 1 describes the data sources along with the respective sensors, data access methods, data types, and sampling rates. The Looxid Link device is mounted on the HTC VIVE Pro Eye device, and it cannot be used at the same with the VIVE Facial Tracker because the HMD has only one connection port for add-on hardware. In addition to enriching embodiment, we plan to use the collected data for research purposes in the future, for example to investigate what physiological effects a VR experience has on users.

### 3.2. Architecture

**Figure 1:**
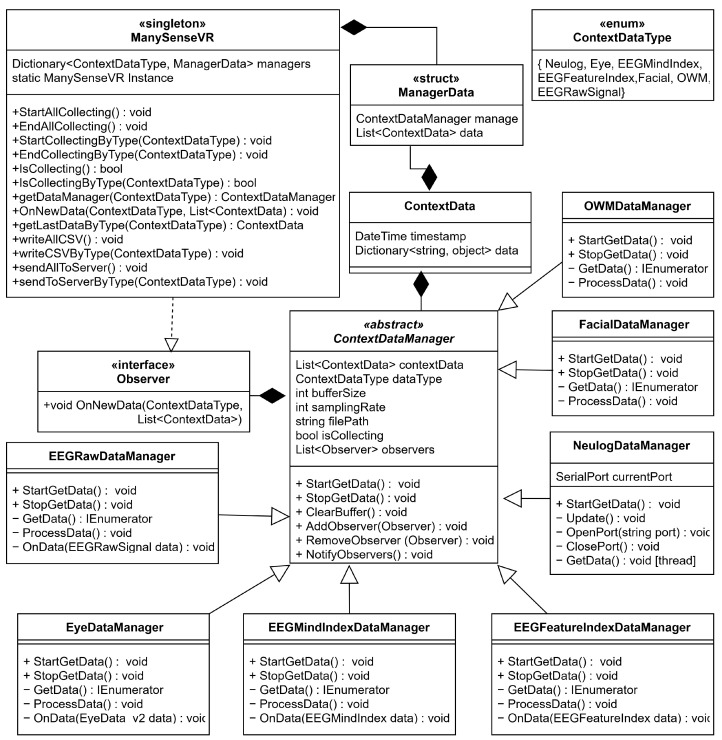
Architecture of ManySense VR.

In the architecture of ManySense VR (Figure 1), the ManySenseVR class is responsible for managing a collection of ContextDataManagers which orchestrate the data collection from different data sources. It has methods for starting and stopping data collection from all or specific types of ContextDataManagers, checking the status of data collection, writing data to a comma-separated values (CSV) file, and sending data to the server. The developer can choose which ContextDataManagers to use by registering them to the dataManagers dictionary of ManySenseVR. The registered managers are then associated with a list of ContextData objects which are received from the ContextDataManagers.

ContextDataManager is an abstract class that defines the common state and behavior for all data collection managers. It contains a contextData list, which is used as a buffer for incoming data. The maximum size of the contextData list is determined by the BufferSize variable, which can be adjusted for each manager. When the buffer is full, it is cleared by moving the data to appropriate ManagerData object held by ManySenseVR. Further, the data in the ManagerData object can be written into a CSV file or sent to a server upon request. ContextDataManager contains common fields such as the type of context data for which the manager is responsible, desired sampling rate, path for storing CSV files, and collection status. Common methods include starting and stopping data collection, and clearing the buffer. Additionally, ContextDataManager has methods that implement the observer design pattern, thus allowing any Observer object to receive data when the buffer is emptied. When ContextDataManagers are registered to ManySenseVR, the latter becomes an observer; this way ManySenseVR can automatically receive data updates from the managers through the callback method OnNewData().

The framework can be extended by inheriting from the ContextDataManager class, thus creating a new manager that connects to a sensor API or another data source to acquire data. The new context data type must also be added to the ContextDataType enumeration, which is used to identify the managers. New managers can specify a buffer size, which determines the rate at which the data is sent forward. Similarly, the developer can set a desired sampling rate and attempt to acquire data at this rate; this can be performed by a Unity coroutine or a thread, for example. The subclasses of ContextDataManager must override StartGetData() because different context data sources require different access methods. The default implementation of StopGetData(), which stops all coroutines and clears the buffer, can be overridden as needed. The subclasses of ContextDataManager are described in the following sections.

Both ManySenseVR and ContextDataManager extend Unity’s MonoBehavior class. Consequently, the developer can attach these components to any Unity scene and thereby leverage Unity’s lifecycle methods. For example, a developer of a new ContextDataManager can override the Update() method of MonoBehavior to perform actions once per frame. Similarly, when these scripts are attached to a Unity scene, their visible properties can be managed with the Unity Editor’s Inspector tool. Moreover, ManySenseVR is implemented using the singleton pattern so it can be accessed through any script in the project. ManySense VR also contains the Framework Launcher helper script that takes the responsibility of configuring the data sources for data collection and starting/stopping ManySense VR upon request.

#### 3.2.1. Eye Data Manager

Referring to Table 1, HTC VIVE Pro Eye HMD has an eye-tracking sensor that produces diverse data related to eye gestures. The EyeDataManager class uses the SRanipal SDK to acquire eye data. The maximum sampling rate of the eye tracker is 120 Hz, but this can be adjusted based on the desired sampling rate. When StartGetData() is called (see Figure 1), EyeDataManager starts the GetData() coroutine—a commonly used concurrency technique in Unity—that processes incoming data and manages the frequency of data sampling based on the sampling rate. To collect data from the eye tracker, the SRanipal engine should be in a working state. After confirming that the engine is available, EyeDataManager registers a callback function OnData() to the SRanipal engine to automatically receive data. The received data is then processed by ProcessData() according to the set sampling rate by the coroutine. In this method, eye activation data is recorded first. After that, various types of eye data—27 items related to openness, pupil size, blinking, eye position, expression, and gaze target—are extracted and stored to a temporary data structure. Once all data in the sample has been processed, the temporary data structure is written into a buffer. When the buffer is full, the data in it are written into a ManagerData struct stored in ManySenseVR.

#### 3.2.2. EEG Data Managers

The Looxid Link EEG sensor can be attached to the HTC VIVE Pro Eye HMD using a USB-C cable. When the user wears the HMD, the EEG electrodes touch the forehead of the user. We used the Looxid Link Unity SDK to collect data, and distributed the EEG data collection process to three ContextDataManagers based on the type of data they provide: EEGRawDataManager (6 data items), EEGFeatureIndexDataManager (42 data items), and EEGMindIndexDataManager (5 data items). This division was performed for two reasons: (i) the SDK provides different data at different sampling rates; and (ii) the data types provided by the SDK are logically different. Basically, all of the EEG data managers collect data in a similar way with EyeDataManager. After StartGetData() subscribes to an appropriate callback method provided by the SDK, the GetData() coroutine starts to sample data based on the sampling rate. Based on the detailed features of the EEG data, the maximum sampling rates are different (Table 1). Like EyeDataManager, the sampling rate can be modified to the desired value in each EEG data manager, and they use buffers for temporary storage of data. When the buffers are full, the data are stored to the appropriate ManagerData struct in ManySenseVR.

#### 3.2.3. Facial Data Manager

The HTC Facial Tracker sensor is attached to the HTC VIVE Pro Eye HMD using a USB-C cable. To capture data from the sensor, the FacialDataManager class uses a similar coroutine-based approach than EyeDataManager. The main difference is that the facial tracker API does not provide a callback method, so FaciaDataManager polls data according to the defined sampling rate. The polled data is inserted into the buffer, and when the buffer becomes full it is written to the appropriate ManagerData struct stored in ManySenseVR. The captured data includes 39 data items related to jaw, mouth, tongue, cheeks, and sensor status.

#### 3.2.4. NeuLog Data Manager

Most of the data sources presented in Table 1 provide a straightforward data acquisition method through a well-defined API. However, in the case of NeuLog sensors, we faced significant problems using the provided NeuLog API. NeuLog sensors are modular and connected to the computer through a USB cable. These sensors can be accessed through the NeuLog API based on a REST connection over hypertext transport protocol (HTTP). The API allows collecting a fixed amount of data over a period of time, or a single data item at any moment. However, there are significant limits. The method of collecting a fixed amount of data is limited to 65535 samples, which corresponds to approximately 11 min. However, VR contents are often longer than that, thus making this method inappropriate. The second method to collect data is to repeatedly request a single data item, but we found it to be slow (5–10 Hz) when using the REST API due to the response-request nature of a HTTP connection. Because of these issues, we decided to acquire the data directly via serial communication. This was performed by analyzing and improving upon an obsolete Python version of the NeuLog API source code that was found on the company website and third-party code found on GitHub. These source codes informed us about the bytes used in the protocol, and how to convert the bytes to more useful values. The operation of the resulting NeulogDataManager script is as follows. It first scans serial ports for connected NeuLog devices by writing specific bytes and waits for a response. When the given bytes are accepted, a specific answer bytes are read back and the script recognizes the port as the NeuLog sensor port. The script then repeats a data collection procedure that reads and writes bytes according to the protocol. We used a dedicated thread to perform the serial port scanning and the data collection procedure to reduce the load of the main thread.

#### 3.2.5. OpenWeatherMap Data Manager

To demonstrate ManySense VR’s extensibility beyond capturing sensor data, we implemented a context data manager to retrieve weather data from the REST API provided by the OpenWeatherMap service that returns a JSON object containing the request data. The OWMDataManager class implements a coroutine to poll weather data (15 data items including data such as location, temperature, air pressure, humidity, wind information, cloudiness, and visibility) for a given city using the current weather data endpoint of the OpenWeatherMap API (https://openweathermap.org/current, accessed on 13 June 2022). The REST communication is implemented using Unity’s UnityWebRequest and JsonUtility classes. The maximum sampling rate, according to our test, was found to be 6–8 Hz; however, weather data is not updated often so we expect that sampling data at every few minutes suffices for most applications.

## 4. VR Embodiment Implementation

### 4.1. Embodiment Scene

**Figure 2:**
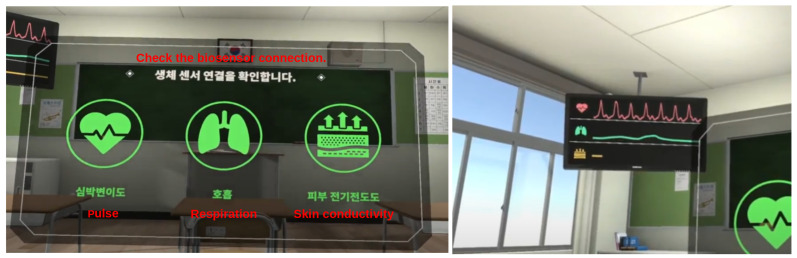
Connectivity indicators for pulse, respiration, and GSR (skin conductivity) sensors with translated annotations (**left**). Sensor data visualization on a virtual television screen (**right**).

We designed an embodiment scene in VR so that the user can feel embodiment through synchronization of their facial expressions, eye movement, and body gestures with the avatar. The scene setting is a classroom environment where the user is seated. We implemented a virtual mirror so that the user can experience embodiment by looking at their avatar through the mirror. The scene also shows a television screen, illustrated in Figure 2, with real-time charts of the user’s pulse, respiration, and GSR data, which aims to further strengthen embodiment.

**Figure 3:**
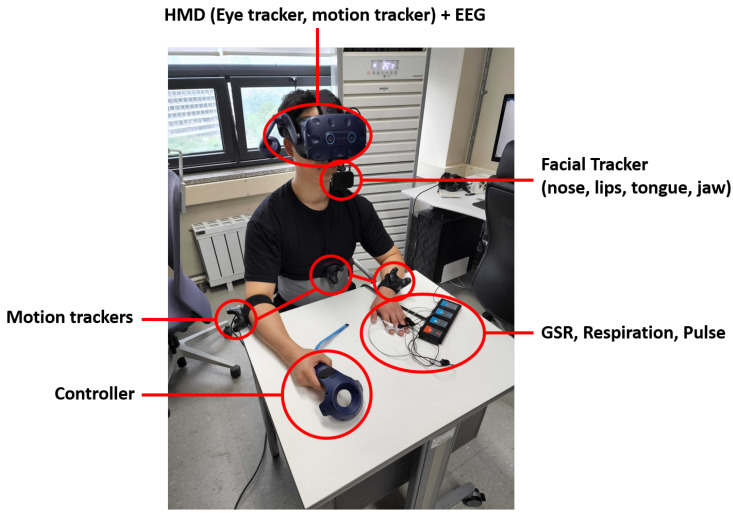
Sensors used in the embodiment scene.

Before entering the VR scene, the user sits down and wears the HTC VIVE Pro Eye HMD, a VIVE Controller, three VIVE trackers, and NeuLog sensors so that the user’s context data related to motion, facial expressions, eyes, and physiology can be collected. The VIVE controller is held in the dominant hand, while the GSR and pulse sensors are worn in the non-dominant hand. The motion sensors are placed on the user’s head, hand, elbow, and waist. Figure 3 depicts a user wearing the devices with annotations for each sensor type.

**Figure 4:**
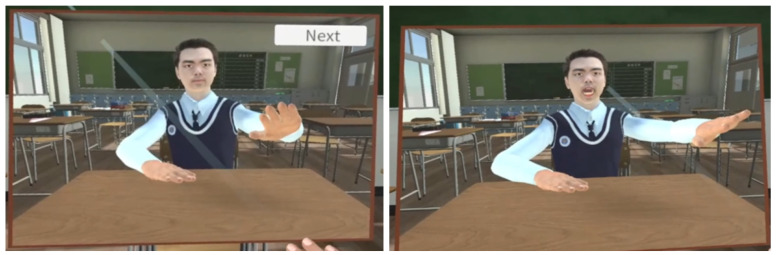
The virtual mirror in the embodiment scene.

When the program starts, the user is placed into the shoes of a virtual avatar who wears a school uniform and sits in a virtual classroom environment. The user can confirm that their movement is synchronized with that of the virtual avatar by moving their head and dominant hand. After showing sensor connectivity information (Figure 2), a virtual mirror appears in front of the user (Figure 4). The mirror shows the virtual avatar’s body, eye, nose, lip, and jaw movement in synchrony with those of the user. We provided the virtual mirror to the user because they cannot otherwise check their avatar’s facial expressions and movement from a first-person perspective in VR. Moreover, to enable real-time embodiment, the virtual mirror should be rendered in real-time. As a solution, we use a virtual camera as a material for the mirror so that the user can see the avatar reacting to their bodily and facial actions in real time.

### 4.2. Motion Embodiment

**Figure 5:**
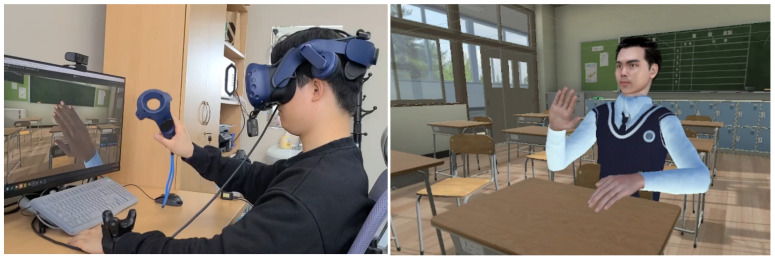
Result of motion embodiment.

**Figure 6:**
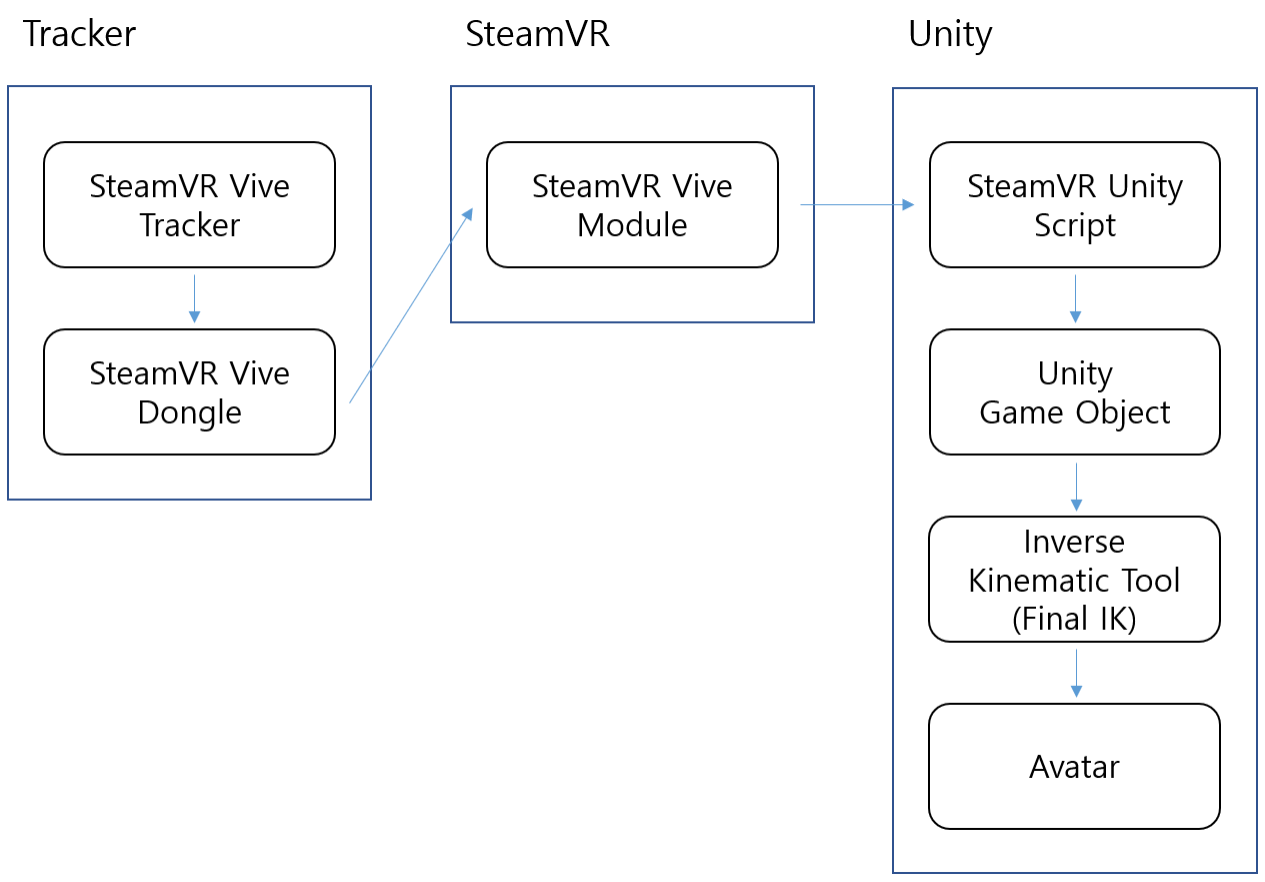
Motion embodiment architecture.

Our motion embodiment implementation uses the motion sensors’ position and rotation data as a reference point for movement. HTC VIVE Pro Eye HMD, VIVE Controller, and VIVE Trackers were used for collecting motion data of different body parts of the user (Figure 3). Using these sensors, we collected the user’s joint transform data that were used to move the avatar’s joints. To achieve this, the SteamVR Unity library was used to convert real-world transform data to Unity transform data. Final IK, an IK animation tool, was then used for moving the virtual avatar according to the captured data. Because it is infeasible to collect data from all joints of the user by attaching motion trackers all over the body, we had to move the avatar using a minimal amount of joint data. Therefore, we choose the IK animation method which calculates the surrounding muscles’ transforms based on a specific muscle. Thus, we connected the trackers only to specific muscles that contribute to key movements, such as hand, elbow, head and waist. As a result, the muscles of the avatar move based on the data provided by the nearest trackers. Figure 5 illustrates the motion embodiment implementation in action, whereas Figure 6 depicts the motion embodiment architecture where motion data is transferred from VIVE Trackers to the avatar model.

### 4.3. Facial Embodiment

**Figure 7:**
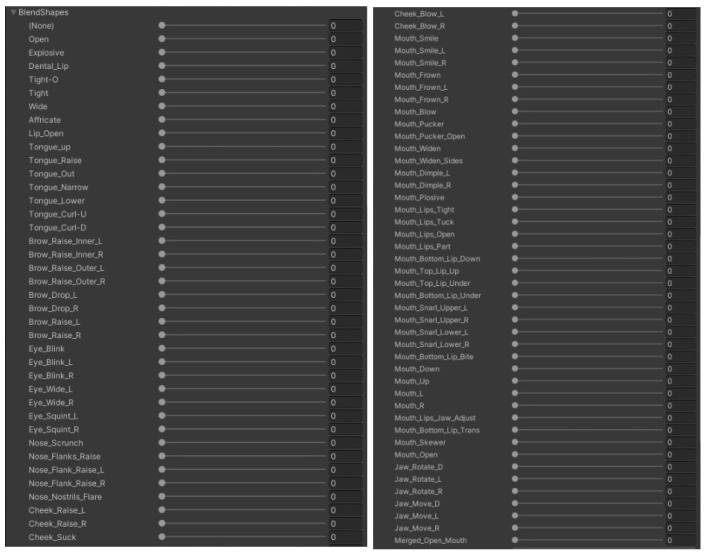
List of blendshapes for the avatar’s face.

**Figure 8:**
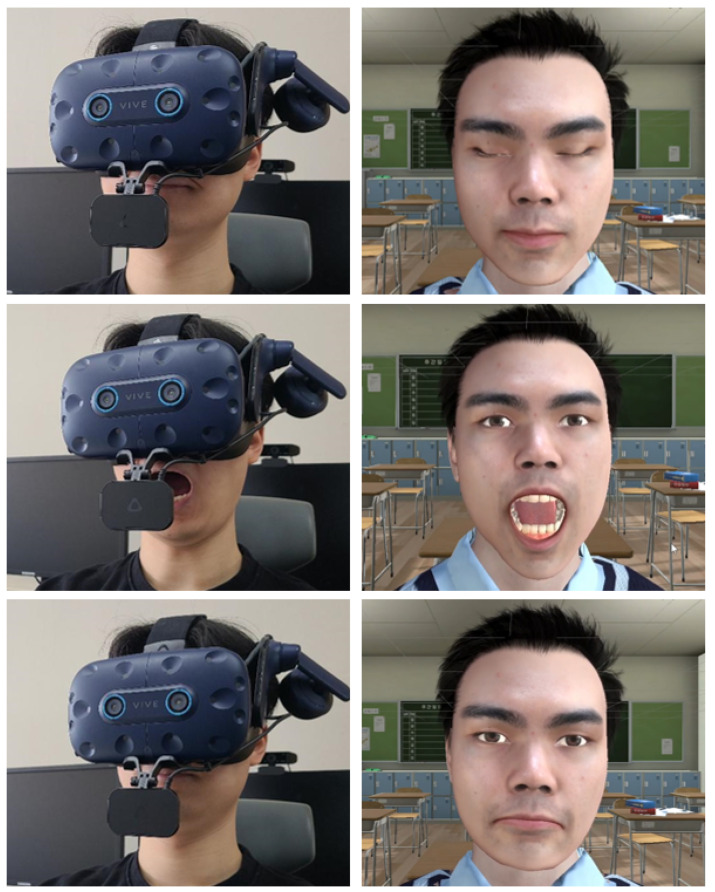
Results of facial embodiment.

The embodiment scene implements facial embodiment by synchronizing the user’s face with the virtual avatar’s face based on facial data. We used the HTC VIVE Pro Eye HMD and HTC VIVE Facial Tracker for capturing eye data and facial expressions, respectively. HTC VIVE Pro Eye captures the user’s upper face expressions, including eyes, and pupils, whereas HTC VIVE Facial Tracker captures lower face expressions including lips, teeth, jaw, and tongue muscle. All captured data were accessed through the SRanipal SDK, which distinguishes each facial muscle based on their position and reads the user’s facial expression. The data were then used to move specific muscles among the muscles on the avatar’s blendshapes (Figure 7) which were made by the IClone software. By adjusting the avatar’s blendshapes, we were able to express its facial expressions in synchrony with those of the user. Figure 8 shows the results of facial embodiment.

### 4.4. Physiological Data Visualization

We used ManySense VR to capture real-time physiological data (pulse, respiration, and GSR) from the NeuLog sensors. The captured NeuLog sensor data are visualized as a real-time graph so as to provide the user with a view of their body state. The graph is displayed on a virtual television object in the embodiment scene (Figure 2). It acquires data from the ManySenseVR class and draws a graph which shows data of the recent five seconds. The graph is updated at every updating interval by obtaining the latest data from ManySenseVR. The updating interval is calculated as (1 s) ÷ (sampling rate) × 10 so that the graph is updated at every 10 samples. The updating interval is about a third of a second when the maximum sampling rate of NeuLog sensors (30 Hz) is used. In drawing the graph, Unity’s Image component is used to represent lines between the points of the graph.

## 5. Performance Evaluation

### 5.1. Performance Evaluation Method

**Figure 9:**
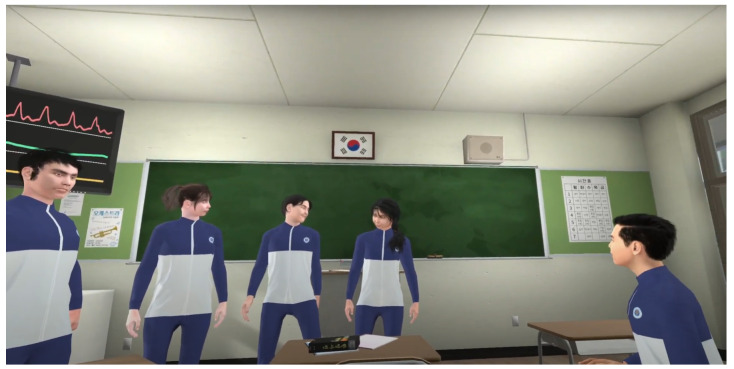
Group scene with animated virtual characters.

The evaluation was conducted by running two different VR scenes and activating different parts of ManySense VR to measure the effect of each manager component. The evaluation parameters are listed in Table 2. The first test scene was the embodiment with a virtual mirror as described above. Because this scene is fairly simple and has no animated virtual characters other than the user’s avatar, we also ran the test with a group scene in which five animated virtual characters interact with each other (Figure 9), thus presumably causing higher system load. In both scenes, we executed tests with six test conditions that collected data from different configurations of data sources. Before running the tests, we terminated all unnecessary processes. During each test, a researcher played through the scenes wearing the HTC VIVE Pro Eye HMD and the sensors described in Table 1. During playing the scenes, we collected statistics (minimum, maximum, mean) on central processing unit (CPU) load, random access memory (RAM) usage, and frame rate in frames-per second (FPS). We repeated each test three times and calculated the mean values of each test metric to minimize the effect of random system load spikes. The buffer size was different for the NeuLog sensors because we needed real-time data for drawing a graph based on the data in the virtual environment. The facial tracker was excluded from test condition six because HTC VIVE Pro Eye device has only one USB port, which was occupied by the EEG sensors. The EEG sensor was prioritized over the facial tracker because the latter uses the same SRanipal library than the eye tracker.

Because Unity Profiler consumes a substantial amount of resources, we wrote custom code to measure FPS (via Time.unscaledDeltaTime in Update()), CPU usage (via .NET System.Diagnostics), and RAM usage (via Unity Profiler API). However, the Vive-Super-Reality-SR_Runtime service process that belongs to the SRanipal SDK is not accessible via .NET System.Diagnostics. In the performance evaluation, this process showed a stable CPU usage between 30% and 34%, with lower usage at the beginning. Therefore, we amended the CPU usage performance test result by 30% when the eye tracker was involved.

### 5.2. Performance Evaluation Results

In the following subsections, we summarize the evaluation results for each test condition and scene on CPU load, frame rate and memory footprint. More detailed data for each test condition are presented in Appendix A, Appendix B, Appendix C, Appendix D, Appendix E and Appendix F.

#### 5.2.1. CPU Usage

**Figure 10:**
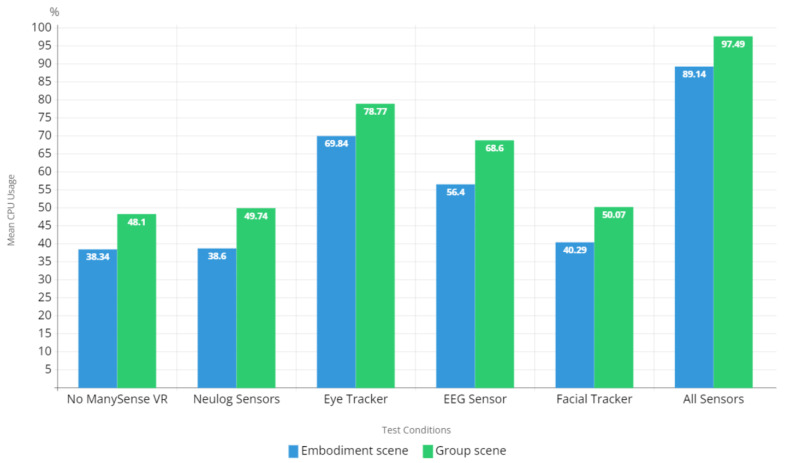
Mean CPU usage (%) for each test condition and scene.

The CPU usage results in Figure 10 show that when new data sources are activated, the CPU usage of the VR content increases, where the extent of the increase depends on the data source. The results indicate that the data managers for NeuLog sensors and facial tracker had little effect on the CPU usage, whereas the data managers for eye tracker and EEG sensor contributed to a greater increase in the CPU usage. In particular, using the eye tracker significantly increased the CPU usage; however, this result includes a 30% CPU load caused by the aforementioned Vive-Super-Reality-SR_Runtime service process. When this service is excluded from the statistics, the mean CPU usage results of ManySense VR with the eye tracker for the embodiment scene and the group scene are 39.84% and 48.77%, respectively. Interestingly, these results are slightly lower than the CPU usage results for test condition 1 (no ManySense VR). Overall, it appears that ManySense VR has little effect on the CPU usage when the SDK routines provided by the sensor manufacturers are not considered.

#### 5.2.2. Frame Rate

**Figure 11:**
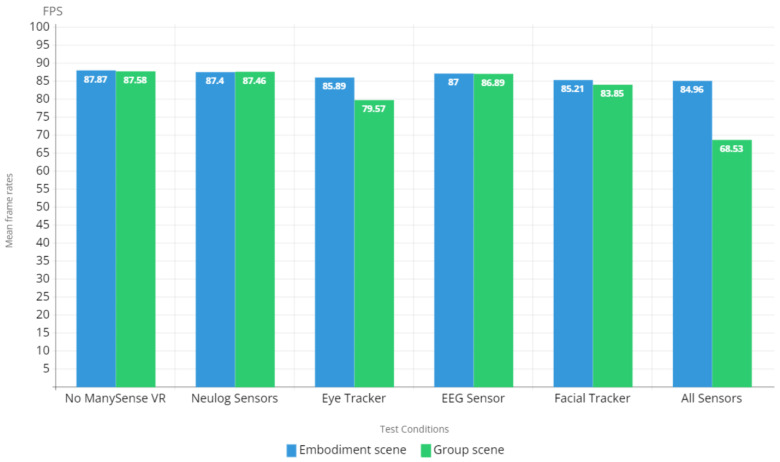
Mean frame rates (FPS) for each test condition and scene.

Sustaining a high frame rate in VR content is essential for facilitating good user experience and for preventing adverse effects such as cybersickness [25,26,27]. The frame rate test results depicted in Figure 11 indicate that the evaluated VR content is capable of producing a mean frame rate of approximately 87 FPS when ManySense VR is not used. The frame rates are similar for most of the test conditions; however, there is a clear drop in the frame rates of the group scene when using eye tracker or all sensors. These results are aligned with the CPU usage results presented in Figure 10; when the CPU usage is 78% or more, the frame rate drops. Therefore, it is important to ensure that the hardware on which the VR content is executed can provide sufficient CPU performance to ensure a high frame rate.

#### 5.2.3. Memory Footprint

**Figure 12:**
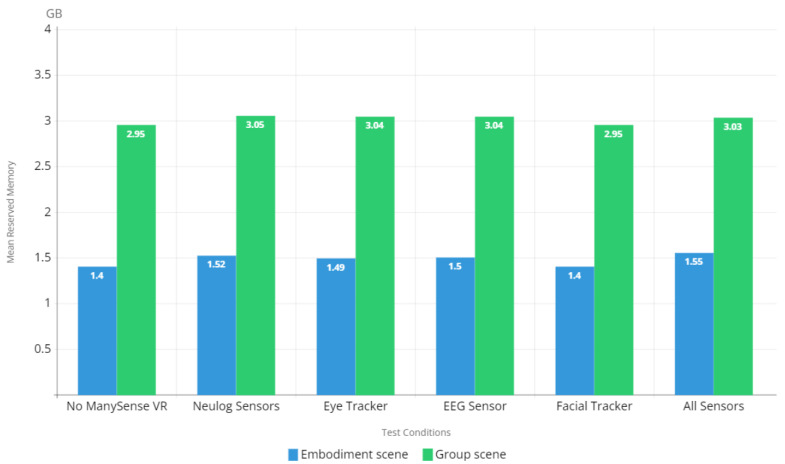
Mean reserved memory (GB) for each test condition and scene.

**Figure 13:**
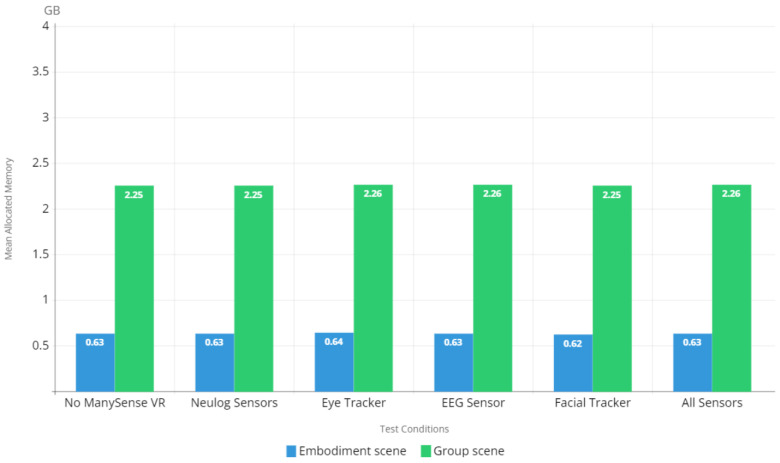
Mean allocated memory (GB) for each test condition and scene.

To investigate the memory footprint of the VR content during the evaluation, we measured the mean reserved memory and mean allocated memory for each test condition and scene. The results regarding reserved memory in Figure 12 show that there is very little overhead on the memory footprint caused by ManySense VR; even when all sensors were used, the difference in reserved memory usage to test condition 1 (no ManySense VR) is negligible. The allocated memory results in Figure 13 show a similar trend, thus confirming that ManySense VR’s overall memory footprint is small.

## 6. Formative Evaluation with Developers

### 6.1. Formative Evaluation Method

The objective of the formative evaluation [28] was to explore perceived advantages, disadvantages, usefulness, ease-of-use, and future prospects of ManySense VR from the perspective of developers, thus providing valuable information to guide future development of the framework. To reach the objective, we used a qualitative research approach where five volunteer university students were interviewed after they performed two implementation tasks related to using and extending ManySense VR. The In the following, we outline the formative evaluation method. As per the decision made by the Institutional Review Board of Ajou University, this experiment was exempted from the ethical review because no personal information was collected nor was the experiment harmful to the participants.

#### 6.1.1. Participants

We recruited five participants (three females and two males; mean age: 22) among undergraduate and graduate students of the Department of Digital Media at Ajou University. The requirement for the participants to join the experiment was to have some experience of Unity development. We accepted any degree of development experience because we wanted to receive feedback both from beginners and more experienced developers. The participants were recruited by advertising at university courses and research laboratories.

#### 6.1.2. Materials

**Figure 14:**
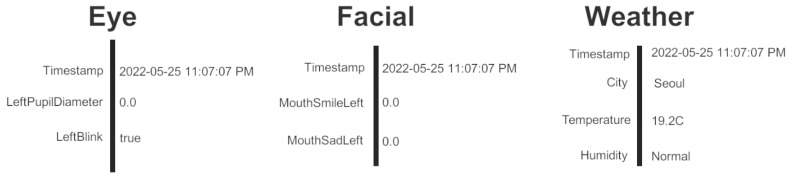
User interface of the skeleton project provided for the participants to update.

**Figure 15:**
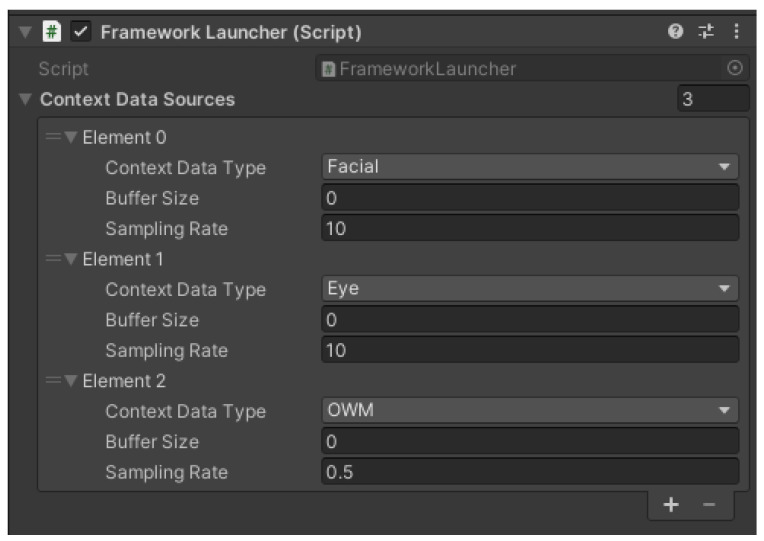
Configuration of the Framework Launcher script in Unity that starts and stops ManySense VR. In this example, the eye tracker, facial tracker, and OpenWeatherMap data managers have been enabled with specific buffer sizes and sampling rates.

To save the participants’ time and to focus on measuring their insights on ManySense VR rather than other aspects of Unity or VR development, we prepared a skeleton Unity project with a pre-configured VR scene that contained a simple user interface comprising labels for presenting data from the eye tracker, facial tracker, and OpenWeatherMap (Figure 14). ManySense VR was imported in the project, but it was not added to the scene. We also prepared two skeleton scripts for the participants to complete as described by two development tasks. The first script, UIUpdater, was to retrieve data from ManySense VR and update the user interface accordingly. The second script was an partial implementation of OWMDataManager that the participant had to complete. The development tasks were as follows:

1. Use ManySense VR
  (a) Add an empty GameObject in the scene and add the Framework Launcher component (Figure 15) to it.
  (b) Configure Framework Launcher to collect data from the eye tracker and facial tracker at specific buffer sizes and sampling rates.
  (c) Complete the UIUpdater script:
    - Retrieve the latest eye tracker data from ManySense VR, following a sample code that queries facial tracker data;
    - Show the left pupil diameter and left blink values of the eye tracker data on the user interface.
  (d) Run the project, start the data collection, and verify that the data is updated on the screen.
  (e) Stop the data collection and verify that the data is automatically written into a CSV file.
2. Extend ManySense VR
  (a) Open and study the OWMDataManager skeleton script that provides the basic structure for a new data manager for the OpenWeatherMap service (detailed explanation was given to the participant in comments).
  (b) Insert the code to the OWMDataManager for retrieving data from OpenWeatherMap, so that it is executed repeatedly in a coroutine.
  (c) Reconfigure the Framework Launcher component to collect data from the OWMDataManager.
  (d) Update the UIUpdater script to query OpenWeatherMap data from ManySense VR and show it on the user interface.
  (e) Run the project, start the data collection, and verify that the data is updated on the screen.
  (f) Stop the data collection and verify that the OpenWeatherMap data is also written into a CSV file.

In addition to the skeleton project, the participants were given a document containing detailed instructions for the development tasks, as well as a spreadsheet containing string keys and descriptions for the data types captured by the eye tracker and the facial tracker (to be used when querying collected data). We also prepared a set of interview questions divided into pre-test and post-test sections. The pre-test questions collected data on demographics (age and gender) and previous experiences on Unity development and data API programming. The post-test questions inquired the participants on their ManySense VR experience, particularly focusing on its perceived advantages and disadvantages for context-aware VR application development, ease of use, comparison with direct data access method, and future prospects as a development tool. All materials were translated to Korean by one Korean researcher and the translations were verified by another Korean researcher.

#### 6.1.3. Procedure

The evaluations were conducted in Korean at a research laboratory at Ajou University in May 2022. The participants joined the one-hour experiment individually. After a participant arrived at the research laboratory, a researcher explained the purpose and the experiment steps to the participant. After that the researcher gave a notification to the participant that the data from the experiment can be used anonymously for research purposes, and received a signature from the participant to the informed consent form. Before the development tasks of the started, the researcher interviewed the participant for demographic information, and their past experiences related to Unity development and programming with data. After the pre-test interview, the development task part of experiment commenced. The whole process of development tasks was recorded by screen recording. After the tasks finished, a post-test interview was conducted by two researchers; one researchers asked the questions while the other researcher took notes. The questions were related to the developer’s experience using the ManySense VR framework. Both pre-test and post-test interviews were recorded, and then transcribed and translated to English.

#### 6.1.4. Data Analysis

The collected interview data were analyzed by an iterative coding process where the participants’ answers were scanned for relevant keywords, and the keywords were then grouped into categories. Observations and screen recordings were analyzed to identify any meaningful behavior and gestures during the development tasks that could complement the interview answers. The interview data were analyzed by two of the authors independently, who then jointly formed the final result set by negotiation.

### 6.2. Formative Evaluation Results

#### 6.2.1. Demographics and Previous Experiences

The demographic details of the five participants, their previous development experiences, and the durations of their experiment tasks are presented in Table 3. Among the participants, Participant 2 was a graduate school student whereas the rest of the participants were undergraduate students. Moreover, as the table indicates, the experience levels of the participants varied significantly, with Participants 2 and 4 being the most experienced Unity developers and Participants 1 and 3 being beginners.

#### 6.2.2. Perceived Advantages, Ease-of-Use, and Efficiency

ManySense VR requires only a few steps for developers to adapt it to Unity projects. The only things that the developers need to do to collect data using ManySense VR are: (i) ensure that the desired data source is available (e.g., sensor devices are powered on and connected to the computer); (ii) attach the Framework Launcher script to a GameObject in a Unity project; and (iii) choose which context data managers to activate in the Framework Launcher options. Optionally, the developer can choose to make a new data manager for custom data retrieval and attach it the framework. The simplicity of these steps was appreciated by the participants, as four of them found ease-of-use in ManySense VR, as the following interview excerpts demonstrate:

“It was easy to get data, so even a beginner could easily follow it.”

(Participant 1)

“It is easy to use if you know the data name.”

(Participant 2)

“It was easy to use.”

(Participant 3)

“It was intuitive and easy to get the data.”

(Participant 5)

In addition to being easy to use, ManySense VR can easily be extended and adapted to meet the developer’s intention. To effectively use ManySense VR, developers can learn and understand the architecture of it. Aligned with the results on ease-of-use, two of the developers found ManySense VR to have good learnability that does not require intensive training:

“After using ManySense VR one time, it seems that users can do many things with it.”

(Participant 1)

“It seems that users can quickly learn and use it.”

(Participant 5)

We observed that Participant 5 attempted to learn and understand the details of ManySense VR’s architecture by studying the framework’s source code during the experiment even though he was not asked to do it. The time allocated to the experiment was too short for the participant to comprehend the inner operations of the framework. This indicates that although learning to use ManySense VR can performed quickly, understanding its internal structure requires time and expertise in Unity. However, such in-depth understanding is not required for most use-cases of ManySense VR.

ManySense VR was developed to be used in Unity. Consequently, the language for the framework is C#, which is the language that Unity uses. Developers with past Unity experience can easily understand ManySense VR’s scripts and can easily find a way to adapt it to their projects. The familiarity with Unity among the participants is a possible reason for them to find ManySense VR easy to use. This familiarity was pointed out by one of the participants, as he found that using ManySense VR is similar to his previous Unity experience:

“It was similar to the existing Unity method, so it was not that difficult as I was familiar with the steps of getting data and showing them in the UI.”

(Participant 5)

In the evaluation with developers, there were two tasks: using ManySense VR and extending it. In the latter task, the participants were asked to extend ManySense VR by collecting weather data using the OpenWeatherMap API. In the first task, the participants were asked to collect context data from the sensor sources attached to the user: the eye tracker and facial tracker. However, in the extension task, OpenWeatherMap was not a sensor data source connected to the user. Therefore, the participants had a chance to experience to use ManySense VR for another type of data source beyond sensors. This advantage – good extensibility – was mentioned by two of the participants:

“It seems that it can be extended using other data sources than sensors.”

(Participant 2)

“Extensibility seems to be good. ”

(Participant 3)

To satisfy the requirements for context-awareness in VR applications with ManySense VR, it was designed to allow collection of context data from diverse sources at the same time. To start or stop collecting the data, to store the collected data to a file or server, and to query collected data in real-time are usual tasks that developers need to implement for acquiring context data. Further, in many cases, context data come not only from one source, but from various sources at once. In ManySense VR, the framework supports those kinds of common functions for the developers to use without implementing the functions separately for each data source. Therefore, the developers can collect data fast and thus save time for analyzing the collected data in their context-aware applications. The fast speed of data retrieval was noticed by one of the participants:

“Using ManySense VR was faster than implementing direct data collection. [...] It was good to get external data (e.g., eye, face, weather) right away.”

(Participant 3)

ManySense VR first stores the collected data into a buffer and then empties the buffer into data dictionary objects (in the ContextData class), which are finally stored to a CSV file or sent to a server. Using the key of a feature, developers can easily query the stored data values from the dictionary to use them in their applications at run-time. In contrast, accessing the same data sources directly is far more complicated as diverse data sources have different connection methods and protocols. Three of the participants found this simplicity in ManySense VR’s data access process in comparison to direct access:

“Using OpenWeatherMap API etc is complicated, but using ManySense VR to collect various types of data at once is simple.”

(Participant 1)

“Direct data access is more complicated to use because there are many other things to be concerned about.”

(Participant 2)

“Compared to the method of collecting data directly, ManySenseVR seems to be much better for not having to implement a query or to know the details.”

(Participant 5)

ManySense VR can be extended to collect arbitrary types of data based on the developer’s needs, such as weather data from the OpenWeatherMap API. In the development tasks, the developers could experience to use ManySense VR with other types of API such as OWM as well as sensor APIs. Consequently, one of the participants mentioned about the richness of context data that ManySense VR can provide:

“It was good not only to import sensor data, but also to use other APIs.”

(Participant 2)

#### 6.2.3. Perceived Disadvantages and Difficulties

The sum of raised disadvantages and difficulties was significantly lower than that of the positive aspects reported in Section 6.2.2. The main concern of the participants was related to the way in which the developer can access the data items after receiving data from ManySense VR; the ContextData class stores data in a dictionary data structure where keys are unique strings representing specific data features provided by a data source. Participant 1 found this method inconvenient to use:

“It was a little inconvenient that we had to know the feature name when receiving the OpenWeatherMap data.”

(Participant 1)

Although the participants were provided with a spreadsheet containing the key strings for the facial tracker and the eye tracker, the spreadsheet did not contain keys for the OpenWeatherMap data manager because the participants were to implement it, thus becoming familiar of the source code and the keys within. The use of string-based keys was also recognized by Participant 2 as a problematic technique, citing an increased probability of human errors when using it:

“It seemed like a lot of people could make string mistakes because data features are retrieved with a string.”

(Participant 2)

The experiment tasks were described in detail in the instructions and did not involve complex problem solving. Therefore, all participants, regardless of their previous experience, were able to complete the tasks within a relatively short time. Consequently, as shown in Section 6.2.2, many of the participants found the framework easy to use. However, Participant 5 pointed out that the framework itself requires time to learn:

“It needs some time to understand the framework architecture and how to use it.”

(Participant 5)

We did not provide full documentation of the framework to the participants because they were expected to follow the instructions to complete the given tasks. However, Participant 5 started to explore the source code of the framework during the experiment although it was beyond the instructions. When asked for a reason for this, he explained that he was looking for ways to access the data, and from his perspective, learning another person’s code can save him from troubles later:

“I was looking for how the data is stored and how to get access to it. I was looking for some methods or some variables that I can use to get data from the framework. [...] If I want to use some feature of another developer, I have to have a deep understanding of the code. If I do not, I could fall into some trouble and waste my time.”

(Participant 5)

The decision of not providing detailed ManySense VR documentation to the participants also affected the interview responses of Participant 2, who expressed that the framework lacks scalability because the data is accessed only by a single method:

“Because data is collected with one function, scalability may be limited from the user’s point of view. It would be nice to add a lot of callbacks.”

(Participant 2)

It must be noted that the context data managers of ManySense VR use the observer pattern that allows developers to register their callback methods to be automatically called when new data is available at the context data manager. Obviously this information should be communicated clearly to developers.

#### 6.2.4. Application Ideas

An important aspect of any tool or framework is that after developers gain basic understanding of it, they can apply the framework in projects to come. To measure the participants’ understanding of ManySense VR’s potential in this regard, we asked them to suggest context-aware application ideas where ManySense VR could be utilized. As a result, three out of five participants gave ideas for future context-aware applications that could utilize the data collection services of ManySense VR in diverse application areas:

“It seems that it could be used for receiving facial data that can be used for treatments, facial recognition applications, recognizing human emotions, and serious games.“

(Participant 1)

“In recruitment interviews, facial expressions are important, so a VR interview could be adapted to facial expressions.”

(Participant 3)

“Visualizing the data collected from a smartwatch like Galaxy Watch or Apple Watch.”

(Participant 5)

Additionally, Participant 2 did not propose any specific future application, but instead gave an idea of pulling data from APIs based on the expected interests of potential users:

“I think we should connect to an API where we can get data from high marketability fields that people are interested in, like stock, Bitcoin, and game data.”

(Participant 2)

Although the proposed ideas are limited by the number of participants, these results indicate that the participants gained some understanding on the purpose and potential of ManySense VR for context-aware application development within a short experiment time. Consequently, these results indicate that ManySense VR can foster creativity of context-aware application developers as they can focus on the application itself rather than data acquisition. If the participants were to explore the framework in depth over a longer period of time and in their own development environment, we expect that more ideas could emerge as they would become familiar with the framework and the other data sources that were not used in the tasks.

#### 6.2.5. Improvement Suggestions

As indicated by Table 3, Participant 2 had more than two years of Unity experience, he gave the highest score (8) for the Unity skill self-report, and needed the shortest time to complete the tasks. Of all the participants, only he gave concrete suggestions on how to improve ManySense VR based on his short experience with it. These suggestions are summarized in Table 4 along with our remarks.

## 7. Discussion

### 7.1. Impacts of the Results

The proposed ManySense VR context data collection framework was created for the purpose of facilitating context-aware VR application development on the Unity platform. The evaluation results showed that ManySense VR had a minor effect on the performance metrics both in the embodiment scene and the group scene. This suggests that ManySense VR can efficiently be used for context-aware VR application development from the perspective of resource use, at least when considering the currently implemented context data managers. Moreover, ManySense VR is loosely coupled in the way that it can be easily attached to any Unity VR project and it is free from any domain-specific relations. Therefore, it could be used for VR application development in various fields. This aspect is further strengthened by the extensibility of ManySense VR; developers having new data sources can, with a reasonable effort, create new context data managers to receive data from the device APIs.

To the best of our knowledge, this study marks the first occasion of proposing a reusable context data collection framework for the purpose of context-aware VR application development. The previous studies on context-aware VR applications that we reviewed [2,5,7,8,16,17] utilized systems that were built for a purpose specific to the research objective. Additionally, these studies did not report any reusable and extensible method or technique for acquiring rich context data that could be then used for context-aware application development. In some cases, quickly developing a system for a specific purpose without considering reusability can be justified. For example, this could happen if a system only needs to access one or two data sources with little or no future data source needs. However, in many cases it is difficult to know how the future needs of the system will evolve when new potentially useful devices emerge in the market. Therefore, although developing a framework such as ManySense VR requires more effort than building a data collection system for one-time use, it can be a worthwhile long-term investment.

Regarding the purpose ManySense VR, we aimed to reduce the difficulty of context-aware VR application development. In context-aware VR application development, several types of highly skilled professionals are required, such as programmers, 3D modelers, data analysts, and others. To develop a VR application that is aware of the user’s context, using physiological sensors is required; however, many developers may not be experienced with using sensors and algorithms needed for context-awareness, thus obtaining context data from diverse sources can be challenging for them. Therefore, expertise on handling data sources and developing context-awareness algorithms is required. The development cost of context-aware VR applications is likely to be higher than development cost of similar VR applications that are context-agnostic. ManySense VR aims to help reduce this cost by providing easy data source connectivity and real-time data that can be used to implement rich context-awareness in VR applications. The formative evaluation results suggested that this aim can be achieved when sufficient instructions and examples are given to developers who are new to ManySense VR. Moreover, it is relatively easy for developers to extend the framework with new data sources as per their project requirements. Consequently, ManySense VR can be useful for VR application developers by reducing difficulties in acquisition of relevant context data. Furthermore, in our future study, we are planning to extend ManySense VR to include useful algorithms to analyze the user’s context data to produce higher level information about the user, such as their emotions, attention level, empathy expression, and more.

### 7.2. On Performance Evaluation Results

The CPU performance test results in Figure 10 revealed that the CPU load increased significantly when eye tracking data was collected. Our further exploration on the matter revealed that the Vive-Super-Reality-SR_Runtime service process that belongs to the SRanipal SDK was responsible for about 30% to the total CPU load. We faced a similar performance issue affecting sampling rate with the official API for accessing the Neulog sensors over HTTP, as described in Section 3.2.4. These examples demonstrate that data access tools provided by sensor device manufacturers may cause performance bottlenecks that are beyond our control. Potential solutions to mitigate these bottlenecks include reducing the sampling rate, upgrading computer hardware, reverse engineering APIs to optimize data collection, and communicating with manufacturers to report identified bottlenecks.

The results in Figure 11 indicated that the frame rate of the VR content in the evaluation fluctuated between 80 and 87 FPS. This is slightly below the recommended 90 FPS, which is required for high VR user experience [25]. Low frame rate is known to induce cybersickness [26] which has been shown to negatively affect the sense of presence in VR [27]. Although the frame rates recorded in the evaluation fall below the recommended FPS, the VR content does not include locomotion of the user, for which a low frame rate would be particularly harmful. Moreover, the VR content has not been optimized; especially the group scene contains multiple animated characters and under-the-hood processing that could be optimized further to increase the frame rate. Overall, based on the results we can conclude that ManySense VR with the currently implemented data managers does not significantly affect the frame rate.

The results of memory footprint analysis (Figure 12 and Figure 13) showed very little difference between test condition 1 (no ManySense VR) and test condition 6 (all sensors). This can be partly attributed to the buffering used in ManySense VR; collected data is kept in the buffer only for a short period of time after which the data in the buffer are moved to a persistent storage (CSV file or database) and the buffer is emptied. In the evaluation, we used the buffer size of 100 for all sensors except NeuLog sensors, for which the buffer size was zero. These relatively small buffers partly explain the low memory footprint. If the buffer size is significantly increased, the memory footprint is consequently expected to grow. Moreover, the memory footprint evaluation results suggest that the libraries provided by the eye tracker and EEG sensor manufacturers have little impact on memory footprint, thus confirming their essential operation as data brokers which pass data from the hardware to the client.

### 7.3. On Formative Evaluation Results

The formative evaluation results showed that ManySense VR is easy to use when the participants have access to sufficient instructions and examples, regardless of the participants’ previous Unity experience level. The participants also cited the framework’s learnability and simplicity in comparison to the prospect of directly accessing data source APIs. One likely factor to contribute to this result is that ManySense VR was implemented on Unity, which is popular among the students at the Department of Digital Media at Ajou University. This similarity aspect was brought up in the experiment by Participant 5. The familiarity of a development platform not only reduces the learning curve of a new tool based on the platform, but it is also likely to incentivize learning of the tool because the users can expect use the tool in their future projects.

We found that providing a detailed documentation of the framework’s inner workings can be beneficial for experienced Unity developers, such as Participant 2 and Participant 5, who may want to gain a deep insight of the tool they are using. However, also less-experienced developers could benefit from learning the framework’s operating principles, as it would help them understand the capabilities and limitations of the framework. To facilitate this learning process, easy-to-approach documentation and tutorials would need to be created, such as YouTube videos explaining the framework’s source code and how to extend it.

The suggestions to improve the framework and use it in a variety of applications provided us with solid ideas for development work. For example, Participant 2’s suggestion of adding more callback methods to retrieve data is on our to-do list; more callback methods will be added to the observer pattern, which will be parametrized to give the developer more freedom and flexibility in choosing what data to receive, how and when, and whether the data needs to be preprocessed by filters. Similarly, it is important to support different data formats so that an application that uses ManySense VR to collect data can easily pass the data to a third-party component, such as a server, for further storage and processing.

Participant 2 suggested to add a method to monitor incoming data during data collection. This feature suggestion is valuable, as it would allow implementation of different monitoring strategies, such as receiving monitoring data at certain intervals, receiving alerts when data source errors occur (e.g., sensor disconnection or API timeout), and receiving alerts on data errors (e.g., the data value is out of acceptable range). Such a monitoring system can be coupled with existing storage and visualization tools, such as ElasticSearch, which is already used on our server.

### 7.4. On Richness of Embodiment

By experiencing the virtual environment in the body of the virtual avatar, the user can feel the sense of self-location, sense of agency, and sense of body ownership, which are subcomponents of the sense of embodiment [10]. The proposed embodiment implementation that is based on various sensor devices supports, to some extent, all these subcomponents. The sense of self-location refers to the spatial experience of being inside a body [10] and our embodiment scene aims to convey this experience through a synchronized mirror image of the avatar. According to Kilteni et al. [10], the sense of agency manifests itself through active movements and motor control. In VR this means that the user’s predictions of the outcomes of their movement should be met in the movement of the avatar. Our embodiment implementation provides near-real-time synchronization of the user’s upper body movement including also facial details; however, as legs and fingers are not tracked, the sense of agency is not fully supported. The sense of ownership means that the user feels that the body is their own [10]. A famous example of this is the rubber hand illusion where the participant’s mind is tricked to believe that the rubber hand is their own by synchronously stroking both real and rubber hands with a paintbrush [29]. As Kilteni et al. [10] pointed out, the sense of ownership can diminish if there is discrepancy between the user’s real body and the virtual body. Although we used a rich configuration of sensors to synchronize body actions of the user to those of the avatar, the avatar’s facial appearance did not resemble the face of the user, which may negatively affect the sense of ownership. This is something that we intend to improve in the future, as a previous study has shown that when an avatar’s face matches that of the user, the user’s attention level increases along with the feeling of identification [30].

### 7.5. Personalized User Experience

Data provided by ManySense VR can be useful for personalizing the VR experiences. For example, a relaxation VR experience for stress recovery [31] could be enhanced by adjusting the content based on GSR, heart rate, and other context data. As another example, collected context data could be used to develop a measurement of the user’s empathy expression level by analyzing cues such as eye contact, leaning forward, body orientation, and distance [32]. Similarly, by detecting emotions from context data, we can develop VR systems that respond to the user’s emotional state [5]. Moreover, centralized collection and storage of context data is also useful for researchers who wish to understand the user experience or usability of a VR experience [33]. In general, by analyzing the collected data, researchers can develop new prediction models based on machine learning and deep learning, which can then be used for implementing artificial intelligence-based features, such as virtual characters that perform human-like communication, in future VR applications [34]. In conclusion, the proposed ManySense VR context data collection framework enables rich possibilities for personalized VR applications whilst allowing extension toward future context data sources.

### 7.6. Future Prospects

Although the original motivation for developing ManySense VR was to facilitate collection of context data from sensor sources for the purpose of embodiment, our experimentation with the Open Weather Map API shows that the framework can be used for acquiring data from other sources beyond sensors. Therefore, in the future we plan to develop more context data managers that fetch data from online APIs to be used in VR application for experience personalization. Consequently, VR applications could increase their awareness of the user’s context that lies beyond their body. This approach is similar to the concept of virtual sensors that cover both fusion sensors that combine data from multiple sources, and external data sources such as web services and other online data repositories [11]. By extending ManySense VR toward virtual sensors, its ability to support context-aware applications would increase along with its theoretical maximum resource use. However, we do not expect the latter to be a problem in practice because the developer can choose which context data managers to activate for their application, and most applications have a limited number of required context data sources.

The previously proposed ManySense middleware for Android devices was not only able to collect sensor data but also perform context inferencing on the collected data [11]. The latter is currently missing in ManySense VR, but it is an idea that is worth exploring given that previous research has shown lack of awareness to be a prevalent issue in context-aware middleware systems [6]. For example, a developer could use such a context inference manager to request data about the user’s current emotional state based on an EEG-based emotion classification model [35] rather than acquiring EEG sensor data and performing analysis. This would enable developers unfamiliar with context data analysis to leverage high level context information in their applications. Some sensor device APIs, such as Looxid Link, already provide access to high level context information based on analyzed raw data; however, the application developer still needs to connect to all the separate APIs of different devices to pull out the required data.

Although our evaluation did not find any significant performance issues with ManySense VR, it is possible that the framework has an impact on the VR application performance as the number of active context data managers grows and their overall complexity increases through the inclusion of context inference modules. One solution to this issue is to offload some of the context data managers, particularly heavy context inference tasks, to a dedicated process or even to another machine. These offloaded context data managers would then communicate with the VR application as well as the storage server, at appropriate time intervals, to disseminate the collected or inferred data.

We created and evaluated ManySense VR in the context of context-aware VR application development because many of the implemented context data managers connect to sensors that are attached to the VR HMD (eye tracking, EEG, facial tracker). However, given that Unity supports multiple hardware and software platforms, ManySense VR could be used for adding context-awareness to non-VR applications, such as computer and mobile games. Among the currently implemented context data managers only NeulogDataManager and OWMDataManager are usable in non-VR applications, thus new context data managers need to be developed. From the developer’s perspective, using ManySense VR in a Unity project is similar to using any other Unity component: the Framework Launcher component can be added to a GameObject in a scene and then configured in the Unity editor according to the desired data collection goal. When the scene is executed, the Framework Launcher automatically starts with the GameObject that contains it. This basic method of using GameObjects and components is valid for both VR and non-VR Unity projects. Although ManySense VR appears to be usable outside VR applications, it has not been verified yet in a non-VR project; this is an aspect to explore in a future development study.

### 7.7. Limitations

The study and its results have the following limitations that should be considered. First, although ManySense VR aims to flexibly capture context data from diverse data sources, there are known hardware limitations regarding compatibility of devices, which consequently limit the potential use cases of ManySense VR. For example, the VIVE Facial Tracker and the Looxid Link EEG sensor cannot be used at the same time because they both must connect to the single USB port of the HTC VIVE Pro Eye HMD. Second, this study focused on the technical description of ManySense VR, the embodiment implementation, performance testing, and formative evaluation. We did not consider user experience aspects, so the effects of the embodiment scene implementation remains to be explored in a future study. Third, the performance of ManySense VR was only evaluated in the context of one VR application with only a few context data managers implemented, which limits the generalizability of the results. We aim to add more context data managers to ManySense VR and implement different context-aware VR applications based on the framework as future development work. Lastly, although the formative evaluation results were satisfactory in terms of providing us with valuable information on the framework from the developers’ perspective, the sample of five undergraduate and graduate students lacked depth and diversity. A larger sample of developers from different areas (e.g., education, research, gaming) could yield more information to guide the future development of ManySense VR.

## 8. Conclusions

Developing context-aware VR applications can be challenging as diverse context data sources require different data collection approaches. This development workload can be significantly decreased through code reuse. In pursuit of this objective, we proposed ManySense VR, a reusable and extensible context data collection framework for Unity that can be leveraged by VR application developers to easily capture multimodal context data. We also developed a rich embodiment scene with a virtual mirror where ManySense VR was used to collect context data for the purpose of constructing a virtual avatar that responds to the user’s bodily actions and facial expressions. ManySense VR was then evaluated in the embodiment scene along with a group scene. The results revealed that the framework and its context data managers have little performance overhead in terms of CPU usage, frame rate, and memory footprint; however, the external SDKs used for capturing context data can significantly affect the CPU usage and frame rate, as seen when eye tracking data was captured. Moreover, the results of the formative evaluation with developers indicated that the framework is easy to use and provides a fast method for acquiring data from different sources. The developers participating in the evaluation pointed out extensibility, simplicity, learnability and familiarity as the key advantages of ManySense VR. Moreover, they cited difficulties regarding data querying using string keys and lack of callback methods to access the captured data. Finally, the participants gave application ideas and improvement suggestions as potential directions for future development of ManySense VR. Based on the findings of the performance and formative evaluations, we conclude that ManySense VR has potential to be used as an efficient tool to collect data for context-aware VR applications created with Unity.

The embodiment scene described in this paper uses multimodal data sources to synchronize the avatar and the user. However, there are aspects to be developed to increase context-awareness, such as implementation of hand and finger gestures through camera-based hand tracking or haptic gloves, implementation of lower-body embodiment, and utilization of EEG data to understand the user’s internal state (e.g., emotions and attention). As this study focused on developing a data collection framework, we leave the development of context-awareness to future work. As other future work, we plan to implement more context data managers in ManySense VR, including but not limited to open REST APIs, body trackers, and environmental probes. These will consequently enable richer context-awareness in VR applications, which, in turn, facilitates a higher degree of immersion in future metaverse environments.

## Figures and Tables

**Table 1:** Context data sources used in the study (OWM: OpenWeatherMap).

Source	Source Type	Data Access Method	Data	Max Hz
HTC VIVE Pro Eye HMD	Eye Tracker	SRanipal SDK (Unity)	Gaze direction; Eye expression (frown, squeeze, widen); Pupil movement and size; Eyelid movement (openness, blinking)	120 Hz
	SteamVR Tracking	SteamVR	Position, orientation	90 Hz
VIVE controllers	SteamVR Tracking	SteamVR	Position, orientation	90 Hz
VIVE trackers	SteamVR Tracking	SteamVR	Position, orientation	120 Hz
Looxid Link	EEG (AF3, AF4, Fp1, Fp2, AF7, AF8)	Looxid Link Unity SDK	Raw EEG data	100 Hz
			Feature Index (alpha, beta, delta, theta, gamma, band power)	10 Hz
			Mind Index (attention, asymmetry, relaxation, brain activity)	10 Hz
VIVE Facial Tracker	Facial Tracker	SRanipal SDK (Unity)	Facial expression (nose, lips, tongue, jaw)	60 Hz
NeuLog	Pulse (wave, BPM)	Serial communication	Heart rate	30 Hz
	Respiration		Value of pressure by respiration	
	Galvanic Skin Response		Skin conductivity	
OWM	Current weather	REST API	Temperature, pressure, humidity, wind, rain, snow, clouds, etc.	6–8 Hz

**Table 2:** Evaluation parameters.

Evaluation Parameter	Value
Computer hardware	Intel Core i5 11400, Samsung DDR4 16 GB, NVIDIA Geforce RTX 2060
Operating System	Windows 10 (Version: 10.0.19042, Build: 19042)
VR content execution environment	Unity Editor 2020.3.26f1
Scene 1	Embodiment scene
Scene 2	Group scene
Test condition 1	No ManySense VR
Test condition 2	NeuLog sensors
Test condition 3	Eye tracker
Test condition 4	EEG sensor
Test condition 5	Facial tracker
Test condition 6	All sensors
Buffer sizes	NeuLog sensors: 0 samples, other data sources: 100 samples
Sampling rates	According to Table 1.
Measured values	CPU usage, memory footprint, frame rate (FPS)

**Table 3:** Demographics, previous development experiences, and task duration results of the participants.

No.	Gender	Age	Unity Skill (1–10)	Unity Experience (Time)	Unity Experience (Projects)	Data API Experience	Tasks Duration (mins)
1	F	20	2	<6 months	AR/VR game development.	None	29
2	M	25	8	>24 months	2D games, puzzle games, simulation game platform development.	Showing stock data from an API and making a recommendation list using machine learning.	15
3	F	21	3	<6 months	VR classroom development.	Data visualization using the OWM API.	30
4	F	23	3	>12 months	UI part of an empathy type diagnosing VR application.	Web development project with user data in the server part using a REST API.	23
5	M	21	6	>24 months	VR immersive media programming contents, simple games.	Web crawling, using the OWM API	47

**Table 4:** Improvement suggestions from Participant 2.

Suggestion	Remark
[on strings being used as keys in data retrieval] “I think it would be better to query the dictionary using enums instead of strings. Or, even if it is queried with strings, it would be better from the user’s point of view to make the strings constants.”	This suggestion is aligned with the critiques of Participant 1 and Participant 2 on the use of arbitrary strings as keys for querying specific data features (Section 6.2.3). We agree with this suggestion and plan to improve this feature in a future version of ManySense VR.
“It would be better if the framework could let the developer define the desired data format, since it is currently stored as CSV.”	In the experiment, the data was set to be written in a CSV file on the local machine. Additionally, ManySense VR also supports sending data to a server using JSON format; however, in the current implementation the communication code with the server is fixed to a certain server used in our project. This should be improved in the future to allow the developer to choose from multiple communication methods or even add their own. The same approach should be used for local file storage so that also other formats except CSV could be supported.
“It would be nice to add a lot of callbacks. If you need to collect detailed data, or if a problem occurs, you should be able to change the [data collection] function.“	This suggestion was given in conjunction with the critique of having a single data access method (Section 6.2.3). Although ManySense VR provides data access via the observer pattern, we agree with Participant 2 in that there could be more different types of callback methods; these are elaborated in Section 7.3.
“It would be nice if the user could check the data in detail with a callback every few seconds.”	This suggestion was given when the participant and the interviewer were discussing about the lack of data monitoring during data collection. Currently, the only way for a developer to monitor the data during data collection is to build a monitoring system by themselves. We therefore agree with Participant 2 on the need of this feature in the framework.

## Data Availability

The evaluation dataset is available in Appendix A. Moreover, detailed information on ManySense VR is available on request from the corresponding author.

## Abbreviations

The following abbreviations are used in this manuscript:

API	Application Programming Interface
CPU	Central Processing Unit
CSV	Comma-Separated Values
EEG	Electroencephalogram
FPS	Frames Per Second
GB	Gigabytes
GSR	Galvanic Skin Response
HTTP	HyperText Transport Protocol
IK	Inverse Kinematics
RAM	Random Access Memory
REST	REpresentional State Transfer
SDK	Software Development Kit
VR	Virtual Reality

## Appendix A. Test Condition 1: No ManySense VR

**Table A1:** Results for Test Condition 1: no ManySense VR. The columns show the results for each test run.

	Embodiment Scene 1	Embodiment Scene 2	Embodiment Scene 3	Group Scene 1	Group Scene 2	Group Scene 3
Mean CPU Usage	38.59%	38.39%	38.05%	46.10%	48.32%	49.90%
Minimum CPU Usage	34.11%	34.90%	32.81%	39.84%	42.45%	44.53%
Maximum CPU Usage	61.46%	63.28%	64.32%	65.10%	66.41%	65.89%
Mean FPS	88.74	87.48	87.40	87.80	87.10	87.86
Minimum FPS	26.87	27.95	24.95	53.80	56.63	55.58
Maximum FPS	90.40	90.31	90.33	91.05	90.30	90.32
Mean Total Reserved Memory	1.19 GB	1.51 GB	1.51 GB	2.77 GB	3.04 GB	3.04 GB
Min Total Reserved Memory	1.19 GB	1.51 GB	1.51 GB	2.77 GB	3.04 GB	3.04 GB
Max Total Reserved Memory	1.19 GB	1.51 GB	1.51 GB	2.87 GB	3.05 GB	3.05 GB
Mean Total Allocated Memory	0.61 GB	0.64 GB	0.64 GB	2.24 GB	2.26 GB	2.26 GB
Min Total Allocated Memory	0.61 GB	0.62 GB	0.61 GB	2.23 GB	2.25 GB	2.25 GB
Max Total Allocated Memory	0.62 GB	0.65 GB	0.65 GB	2.27 GB	2.30 GB	2.29 GB

**Table A2:** Mean results for Test Condition 1.

	Embodiment Scene	Group Scene
Mean CPU Usage	38.34%	48.11%
Mean FPS	87.87	87.59
Mean Total Reserved Memory	1.40 GB	2.95 GB
Mean Total Allocated Memory	0.63 GB	2.25 GB

## Appendix B. Test Condition 2: NeuLog Sensors

**Table A3:** Results for Test Condition 2: NeuLog sensors.

	Embodiment Scene 1	Embodiment Scene 2	Embodiment Scene 3	Group Scene 1	Group Scene 2	Group Scene 3
Mean CPU Usage	38.45%	38.29%	39.08%	49.78%	49.15%	50.32%
Minimum CPU Usage	34.38%	33.59%	34.64%	41.67%	44.01%	43.49%
Maximum CPU Usage	61.20%	66.15%	62.76%	63.80%	64.58%	67.71%
Mean FPS	87.41	87.39	87.43	87.80	87.31	87.29
Minimum FPS	23.94	23.95	24.85	56.67	55.85	55.49
Maximum FPS	90.42	90.23	90.37	90.81	90.54	90.48
Mean Total Reserved Memory	1.52 GB	1.53 GB	1.51 GB	3.09 GB	3.04 GB	3.04 GB
Min Total Reserved Memory	1.52 GB	1.53 GB	1.51 GB	3.09 GB	3.04 GB	3.04 GB
Max Total Reserved Memory	1.52 GB	1.53 GB	1.51 GB	3.19 GB	3.05 GB	3.05 GB
Mean Total Allocated Memory	0.63 GB	0.64 GB	0.64 GB	2.25 GB	2.26 GB	2.26 GB
Min Total Allocated Memory	0.61 GB	0.61 GB	0.62 GB	2.24 GB	2.25 GB	2.25 GB
Max Total Allocated Memory	0.64 GB	0.65 GB	0.65 GB	2.29 GB	2.29 GB	2.29 GB

**Table A4:** Mean results for Test Condition 2.

	Embodiment Scene	Group Scene
Mean CPU Usage	38.60%	49.75%
Mean FPS	87.41	87.47
Mean Total Reserved Memory	1.52 GB	3.06 GB
Mean Total Allocated Memory	0.64 GB	2.26 GB

## Appendix C. Test Condition 3: Eye Tracker

**Table A5:** Results for Test Condition 3: eye tracker.

	Embodiment Scene 1	Embodiment Scene 2	Embodiment Scene 3	Group Scene 1	Group Scene 2	Group Scene 3
Mean CPU Usage	70.05%	69.22%	70.27%	79.17%	78.39%	78.77%
Minimum CPU Usage	65.55%	63.73%	66.07%	74.41%	73.63%	73.36%
Maximum CPU Usage	80.14%	79.09%	79.61%	90.03%	88.47%	90.03%
Mean FPS	85.79	85.97	85.92	78.17	81.73	78.84
Minimum FPS	22.86	25.70	25.73	49.99	58.99	56.33
Maximum FPS	90.57	90.51	90.44	86.40	89.16	85.93
Mean Total Reserved Memory	1.46 GB	1.51 GB	1.50 GB	3.06 GB	3.04 GB	3.04 GB
Min Total Reserved Memory	1.46 GB	1.51 GB	1.50 GB	3.06 GB	3.04 GB	3.04 GB
Max Total Reserved Memory	1.46 GB	1.51 GB	1.50 GB	3.07 GB	3.05 GB	3.05 GB
Mean Total Allocated Memory	0.64 GB	0.64 GB	0.64 GB	2.26 GB	2.26 GB	2.26 GB
Min Total Allocated Memory	0.61 GB	0.62 GB	0.62 GB	2.25 GB	2.25 GB	2.26 GB
Max Total Allocated Memory	0.65 GB	0.65 GB	0.65 GB	2.29 GB	2.29 GB	2.29 GB

**Table A6:** Mean results for Test Condition 3.

	Embodiment Scene	Group Scene
Mean CPU Usage	69.85%	78.78%
Mean FPS	85.89	79.58
Mean Total Reserved Memory	1.49 GB	3.05 GB
Mean Total Allocated Memory	0.64 GB	2.26 GB

## Appendix D. Test Condition 4: EEG Sensor

**Table A7:** Results for Test Condition 4: EEG Sensor.

	Embodiment Scene 1	Embodiment Scene 2	Embodiment Scene 3	Group Scene 1	Group Scene 2	Group Scene 3
Mean CPU Usage	56.58%	55.49%	57.14%	68.85%	68.31%	68.66%
Minimum CPU Usage	52.08%	25.78%	50.26%	63.80%	61.72%	63.80%
Maximum CPU Usage	77.86%	72.92%	76.56%	82.29%	86.98%	82.81%
Mean FPS	87.28	86.50	87.23	86.84	87.40	86.43
Minimum FPS	25.82	76.68	24.79	53.49	51.61	52.71
Maximum FPS	90.59	90.73	90.748	90.86	90.60	90.53
Mean Total Reserved Memory	1.49 GB	1.51 GB	1.50 GB	3.06 GB	3.04 GB	3.04 GB
Min Total Reserved Memory	1.49 GB	1.51 GB	1.50 GB	3.06 GB	3.04 GB	3.04 GB
Max Total Reserved Memory	1.49 GB	1.51 GB	1.50 GB	3.06 GB	3.05 GB	3.05 GB
Mean Total Allocated Memory	0.64 GB	0.63 GB	0.64 GB	2.26 GB	2.26 GB	2.26 GB
Min Total Allocated Memory	0.61 GB	0.61 GB	0.62 GB	2.25 GB	2.26 GB	2.26 GB
Max Total Allocated Memory	0.65 GB	0.63 GB	0.65 GB	2.29 GB	2.30 GB	2.29 GB

**Table A8:** Mean results for Test Condition 4.

	Embodiment Scene	Group Scene
Mean CPU Usage	56.40%	68.61%
Mean FPS	87.01	86.89
Mean Total Reserved Memory	1.5 GB	3.05 GB
Mean Total Allocated Memory	0.64 GB	2.26 GB

## Appendix E. Test Condition 5: Facial Tracker

**Table A9:** Results for Test Condition 5: Facial Tracker.

	Embodiment Scene 1	Embodiment Scene 2	Embodiment Scene 3	Group Scene 1	Group Scene 2	Group Scene 3
Mean CPU Usage	40.37%	40.08%	40.43%	50.43%	49.63%	50.17%
Minimum CPU Usage	36.62%	36.35%	35.31%	43.21%	45.03%	43.73%
Maximum CPU Usage	65.26%	62.14%	64.78%	78.10%	57.53%	60.92%
Mean FPS	85.82	85.46	84.36	84.01	83.23	84.33
Minimum FPS	80.27	78.01	78.59	61.37	47.46	59.54
Maximum FPS	94.07	93.99	92.69	90.88	91.09	91.03
Mean Total Reserved Memory	1.39 GB	1.41 GB	1.39 GB	2.96 GB	2.94 GB	2.94 GB
Min Total Reserved Memory	1.39 GB	1.41 GB	1.39 GB	2.96 GB	2.94 GB	2.94 GB
Max Total Reserved Memory	1.39 GB	1.41 GB	1.39 GB	3.09 GB	2.94 GB	2.94 GB
Mean Total Allocated Memory	0.63 GB	0.63 GB	0.63 GB	2.25 GB	2.26 GB	2.25 GB
Min Total Allocated Memory	0.62 GB	0.62 GB	0.62 GB	2.25 GB	2.25 GB	2.25 GB
Max Total Allocated Memory	0.63 GB	0.63 GB	0.63 GB	2.26 GB	2.28 GB	2.26 GB

**Table A10:** Mean results for Test Condition 5.

	Embodiment Scene	Group Scene
Mean CPU Usage	40.29%	50.08%
Mean FPS	85.21	83.86
Mean Total Reserved Memory	1.40 GB	2.95 GB
Mean Total Allocated Memory	0.63 GB	2.25 GB

## Appendix F. Test Condition 6: All sensors

**Table A11:** Results for Test Condition 6: All sensors.

	Embodiment Scene 1	Embodiment Scene 2	Embodiment Scene 3	Group Scene 1	Group Scene 2	Group Scene 3
Mean CPU Usage	89.03%	89.38%	89.02%	96.88%	97.42%	98.18%
Minimum CPU Usage	83.78%	81.18%	79.35%	83.26%	88.73%	86.13%
Maximum CPU Usage	96.02%	97.06%	95.50%	110.34%	110.60%	115.81%
Mean FPS	84.65	85.23	85.00	68.12	68.25	69.22
Minimum FPS	26.86	23.83	25.89	52.74	43.14	53.64
Maximum FPS	90.72	90.66	90.83	74.59	75.79	75.42
Mean Total Reserved Memory	1.59 GB	1.52 GB	1.54 GB	3.03 GB	3.04 GB	3.04 GB
Min Total Reserved Memory	1.59 GB	1.51 GB	1.54 GB	3.03 GB	3.04 GB	3.04 GB
Max Total Reserved Memory	1.59 GB	1.52 GB	1.54 GB	3.04 GB	3.05 GB	3.05 GB
Mean Total Allocated Memory	0.63 GB	0.64 GB	0.64 GB	2.26 GB	2.26 GB	2.26 GB
Min Total Allocated Memory	0.61 GB	0.62 GB	0.62 GB	2.26 GB	2.25 GB	2.26 GB
Max Total Allocated Memory	0.64 GB	0.65 GB	0.65 GB	2.29 GB	2.29 GB	2.29 GB

**Table A12:** Mean results for Test Condition 6.

	Embodiment Scene	Group Scene
Mean CPU Usage	89.15%	97.49%
Mean FPS	84.96	68.53
Mean Total Reserved Memory	1.55 GB	3.04 GB
Mean Total Allocated Memory	0.64 GB	2.26 GB

## References

[1] Bec A., Moyle B., Schaffer V., Timms K. (2021). Virtual Reality and Mixed Reality for Second Chance Tourism. Tour. Manag..

[2] Emmelkamp P.M., Meyerbröker K. (2021). Virtual Reality Therapy in Mental Health. Annu. Rev. Clin. Psychol..

[3] Hamilton D., McKechnie J., Edgerton E., Wilson C. (2021). Immersive Virtual Reality as a Pedagogical Tool in Education: A Systematic Literature Review of Quantitative Learning Outcomes and Experimental Design. J. Comput. Educ..

[4] Yigitbas E., Heindörfer J., Engels G. (2019). A Context-aware Virtual Reality First Aid Training Application. Proceedings of the Mensch Und Computer 2019, MuC’19.

[5] Seo J., Laine T.H., Oh G., Sohn K.A. (2020). EEG-Based Emotion Classification for Alzheimer’s Disease Patients Using Conventional Machine Learning and Recurrent Neural Network Models. Sensors.

[6] Li X., Eckert M., Martinez J.F., Rubio G. (2015). Context Aware Middleware Architectures: Survey and Challenges. Sensors.

[7] Marín-Morales J., Higuera-Trujillo J.L., Greco A., Guixeres J., Llinares C., Scilingo E.P., Alcañiz M., Valenza G. (2018). Affective Computing in Virtual Reality: Emotion Recognition from Brain and Heartbeat Dynamics Using Wearable Sensors. Sci. Rep..

[8] Gradl S., Wirth M., Zillig T., Eskofier B.M. Visualization of Heart Activity in Virtual Reality: A Biofeedback Application Using Wearable Sensors. Proceedings of the 2018 IEEE 15th International Conference on Wearable and Implantable Body Sensor Networks (BSN).

[9] Biocca F. The Cyborg’s Dilemma: Embodiment in Virtual Environments. Proceedings of the Second International Conference on Cognitive Technology Humanizing the Information Age.

[10] Kilteni K., Groten R., Slater M. (2012). The Sense of Embodiment in Virtual Reality. Presence Teleoperators Virtual Environ..

[11] Westlin J., Laine T.H. (2014). ManySense: An Extensible and Accessible Middleware for Consumer-Oriented Heterogeneous Body Sensor Networks. Int. J. Distrib. Sens. Netw..

[12] Abowd G.D., Dey A.K., Brown P.J., Davies N., Smith M., Steggles P., Gellersen H.W. (1999). Towards a Better Understanding of Context and Context-Awareness. Handheld and Ubiquitous Computing.

[13] Raper J., Gartner G., Karimi H., Rizos C. (2007). Applications of Location–Based Services: A Selected Review. J. Locat. Based Serv..

[14] Raj S., Toporski K., Garrity A., Lee J.M., Newman M.W. (2019). “My Blood Sugar Is Higher on the Weekends”: Finding a Role for Context and Context-Awareness in the Design of Health Self-Management Technology. Proceedings of the 2019 CHI Conference on Human Factors in Computing Systems, CHI’19.

[15] Zhao Y., Jiang J., Chen Y., Liu R., Yang Y., Xue X., Chen S. (2022). Metaverse: Perspectives from Graphics, Interactions and Visualization. Vis. Inform..

[16] Yang J., Sasikumar P., Bai H., Barde A., Sörös G., Billinghurst M. (2020). The Effects of Spatial Auditory and Visual Cues on Mixed Reality Remote Collaboration. J. Multimodal User Interfaces.

[17] Medeiros D., dos Anjos R., Pantidi N., Huang K., Sousa M., Anslow C., Jorge J. Promoting Reality Awareness in Virtual Reality through Proxemics. Proceedings of the 2021 IEEE Virtual Reality and 3D User Interfaces (VR).

[18] Gall D., Roth D., Stauffert J.P., Zarges J., Latoschik M.E. (2021). Embodiment in Virtual Reality Intensifies Emotional Responses to Virtual Stimuli. Front. Psychol..

[19] Krekhov A., Cmentowski S., Kruger J. (2019). The Illusion of Animal Body Ownership and Its Potential for Virtual Reality Games. Proceedings of the 2019 IEEE Conference on Games (CoG).

[20] Kim C.S., Jung M., Kim S.Y., Kim K. (2020). Controlling the Sense of Embodiment for Virtual Avatar Applications: Methods and Empirical Study. JMIR Serious Games.

[21] Gao B., Lee J., Tu H., Seong W., Kim H. (2020). The Effects of Avatar Visibility on Behavioral Response with or without Mirror-Visual Feedback in Virtual Environments. Proceedings of the 2020 IEEE Conference on Virtual Reality and 3D User Interfaces Abstracts and Workshops (VRW).

[22] Wiederhold B.K. (2020). Embodiment Empowers Empathy in Virtual Reality. Cyberpsychol. Behav. Soc. Netw..

[23] Roth D., Latoschik M.E. (2020). Construction of a Validated Virtual Embodiment Questionnaire. IEEE Trans. Vis. Comput. Graph..

[24] Gonzalez-Franco M., Perez-Marcos D., Spanlang B., Slater M. (2010). The Contribution of Real-Time Mirror Reflections of Motor Actions on Virtual Body Ownership in an Immersive Virtual Environment. Proceedings of the 2010 IEEE Virtual Reality Conference (VR).

[25] Kanter D. (2015). Graphics Processing Requirements for Enabling Immersive VR.

[26] Jones M.B., Kennedy R.S., Stanney K.M. (2004). Toward Systematic Control of Cybersickness. Presence Teleoperators Virtual Environ..

[27] Weech S., Kenny S., Barnett-Cowan M. (2019). Presence and Cybersickness in Virtual Reality Are Negatively Related: A Review. Front. Psychol..

[28] Flagg B. (1990). Formative Evaluation for Educational Technologies.

[29] Botvinick M., Cohen J. (1998). Rubber Hands ‘Feel’ Touch That Eyes See. Nature.

[30] Seo Y., Kim M., Jung Y., Lee D. (2017). Avatar Face Recognition and Self-Presence. Comput. Hum. Behav..

[31] Ahmaniemi T., Lindholm H., Muller K., Taipalus T. (2017). Virtual Reality Experience as a Stress Recovery Solution in Workplace. Proceedings of the 2017 IEEE Life Sciences Conference (LSC).

[32] Haase R.F., Tepper D.T. (1972). Nonverbal Components of Empathic Communication. J. Couns. Psychol..

[33] Kamińska D., Zwoliński G., Laska-Leśniewicz A. (2022). Usability Testing of Virtual Reality Applications—The Pilot Study. Sensors.

[34] Hartholt A., Fast E., Reilly A., Whitcup W., Liewer M., Mozgai S. (2019). Ubiquitous Virtual Humans: A Multi-platform Framework for Embodied AI Agents in XR. Proceedings of the 2019 IEEE International Conference on Artificial Intelligence and Virtual Reality (AIVR).

[35] Torres E.P., Torres E.A., Hernández-Álvarez M., Yoo S.G. (2020). EEG-Based BCI Emotion Recognition: A Survey. Sensors.

